# A real-world pharmacovigilance study of FDA Adverse Event Reporting System (FAERS) events for osimertinib

**DOI:** 10.1038/s41598-022-23834-1

**Published:** 2022-11-15

**Authors:** Yanchao Yin, Yamin Shu, Junru Zhu, Feie Li, Juan Li

**Affiliations:** 1 Department of Pharmacy, Tongji Hospital, Tongji Medical College, Huazhong University of Science and Technology, No. 1095 Jiefang Avenue, Wuhan, 430030 China; 2 Department of Cardiac and Vascular Surgery, Tongji Hospital, Tongji Medical College, Huazhong University of Science and Technology, Wuhan, 430030 China

**Keywords:** Cancer, Health care, Medical research, Oncology

## Abstract

Osimertinib was a third-generation, irreversible epidermal growth factor receptor tyrosine kinase inhibitor (EGFR-TKI), which approved by the US Food and Drug Administration (FDA) in 2015 for treatment of non-small cell lung cancer (NSCLC). Our study was to explore the adverse events (AEs) caused by osimertinib through data mining of the US FDA Adverse Event Reporting System (FAERS), and provide reference for clinical safety. Data of osimertinib were collected from the FAERS database covering the period from first quarter of 2016 to the fourth quarter of 2021. Disproportionality analyses was employed to quantify the associated AE signals of osimertinib and detect the risk signals from the data in the FAERS database. Reporting odds ratio (ROR) was used to detect the risk signals from the data in the FAERS database. The definition relied on system organ class (SOCs) and preferred terms (PTs) by the Medical Dictionary for Regulatory Activities (MedDRA). Totally, 9,704,33 reports were collected from the FAERS database, 10,804 reports of osimertinib were identified as the ‘primary suspected (PS)’ AEs. Osimertinib induced AEs occurred in 27 organ systems. 68 significant disproportionality PTs satisfying with the four algorithms were retained at the same time. Unexpected significant AEs such as scrotal volvulus, hepatic function abnormal, venous thromboembolisms might also occur. The median onset time of osimertinib-associated AEs was 58 days (interquartile range [IQR] 14–212 days), and the majority of the AEs occurred within the first 30 days after osimertinib initiation. Our study found significant new AEs signals of osimertinib and might provide support for clinical monitoring and risk identification of osimertinib.

## Introduction

Non-small cell lung cancer (NSCLC) is the leading cause of cancer-related death in the world currently, accounting for about 80–85% of all lung cancers^1,2^. Epidermal growth factor receptor (EGFR) tyrosine kinase inhibitors (TKIs), including gefitinib, erlotinib, and afatinib, are the first-line treatments for NSCLC patients harboring EGFR mutations^3^. However, up to 50% of the patients treated with first-line EGFR-TKIs acquire resistance to these drugs, and acquisition of the T790M mutation is the main mechanism responsible for the progress of resistance^4^. Osimertinib is a third-generation EGFR-TKI, which is suitable for patients with EGFR T790M mutation positive NSCLC whose disease progresses on or after EGFR-TKI therapy^5^. Osimertinib was approved by U.S. Food and Drug Administration (FDA) in November 2015, later in European, Japan and China in 2017. Preclinical studies and phase 1 clinical data from the AURA trial suggested that osimertinib might also be an effective first-line therapy for patients with EGFR mutation-positive advanced NSCLC^6^. Clinical trials have consistently demonstrated superior clinical activity and relative safety of osimertinib in advanced NSCLC patients with EGFR mutations regardless of their EGFR T790M mutation status^7,8^.

Most common adverse reactions of osimertinib (≥ 20%) were diarrhea, rash, dry skin, nail toxicity, and fatigue. Furthermore, the most frequent adverse reactions leading to dose reductions or interruptions were prolongation of the QT interval as assessed by electrocardiogram (ECG), neutropenia, and diarrhea^6^. Based on animal studies, osimertinib caused post-implantation loss, early embryonic death and caused an increase in total litter loss and postnatal death, which may impair fertility in females and males of reproductive potential^7,8^. Because clinical trials are conducted under widely varying conditions, adverse reactions observed in clinical trials may not reflect the real-world situation observed in practice. However, a meta-analysis showed that osimertinib notably increased the risk of cardiac toxicities^9^. Therefore, using data mining algorithm to search for the potential ADRs signals of osimertinib in the real-world is necessary for the study of osimertinib.

FDA Adverse Event Reporting System (FAERS) is a public database which is designed to facilitate the FDA’s post-marketing safety monitoring of drug and therapeutic products, and it is one of the largest pharmacovigilance databases in the world^10,11^. A FAERS study revealed the incidence of cardiotoxicity due to osimertinib compared with other drugs approved by the FDA and also specifically versus other EGFR-TKIs between January 1, 2016, and September 30, 2018, and mainly focused on the cardiotoxicity of osimertinib^12^. Data are lacking regarding the real-world safety of osimertinib from 2019 to 2021. In the present study, we retrospectively excavated and analyzed the AEs of osimertinib by data mining in FAERS from the first quarter of 2016 to the fourth quarter of 2021. Our study results may offer a guide for physicians and health policymakers to monitor ADRs for facilitating the rational use of clinical drugs.

## Results

### General characteristics

The clinical characteristics of osimertinib-associated AEs were described in Table 1. For gender, the incidence of AEs in females (55.45%) accounted for a larger proportion than males. In terms of age composition, patients whose age were over 65 years accounted for a higher proportion (36.98%) than patients under 18 years old and patients whose age between 18 and 65 years old. Non-small cell lung cancer was the most reported indication (49.95%), followed by lung neoplasm malignant (36.98%). US (48.20%) reported the largest number of AEs, followed by Japan (15.93%), China (5.70%), France (3.10%), and Thailand (2.42%). Serious outcomes include death, life-threatening, hospitalization, disability, and other serious outcomes. We divide one of them by all serious outcomes reports, to get the proportion. Death (45.85%) was the most frequently reported serious outcome which might be related to disease progression caused by tumor. Other serious outcomes and hospitalization were reported in 3425 (31.81%) and 1973 (18.33%) cases, respectively. Excluding the unknown reporters, physicians and consumers reported the most AEs in 28.11% and 24.73%, respectively. The number of AEs reported were increasing year by year, and the most reported year was 2021 (31.06%), followed by 2020 (27.90%), 2019 (17.60%), 2018 (10.51%), 2017 (8.10%), and 2016 (4.83%), respectively.

**Table 1:** Clinical characteristics of reports with Osimertinib from the FAERS database (January 2016 to December 2021).

Characteristics	Case number, n	Case proportion, %
Number of events	10,804	
Gender		
Male	3235	29.94
Female	5991	55.45
Unknown	1578	14.61
Age		
< 18	6	0.06
18 ≥ and ≤ 65	2161	20.00
> 65	3995	36.98
Unknown	4642	42.97
Indications (TOP five)		
Non-small cell lung cancer	3881	49.95
Lung neoplasm malignant	2873	36.98
Lung adenocarcinoma	296	3.81
EGFR gene mutation	132	1.70
Neoplasm malignant	121	1.56
Serious Outcome		
Death	4936	45.85
Hospitalization	1973	18.33
Life-Threatening	280	2.60
Disability	142	1.32
Other Serious Outcome	3435	31.90
Reported Countries (Top five)		
America	5208	48.20
Japan	1721	15.93
China	616	5.70
France	335	3.10
Thailand	261	2.42
Reported Person		
Physician	3037	28.11
Consumer	2672	24.73
Health Professional	791	7.32
Other health-professional	445	4.12
Unknown	3459	32.02
Reporting year		
2021	3356	31.06%
2020	3014	27.90%
2019	1902	17.60%
2018	1135	10.51%
2017	875	8.10%
2016	522	4.83%

### Signal detection

Signal strengths reports of osimertinib at the System Organ Class (SOC) level are described in Table 2. According to the statistics, we found that 27 organ systems were involved in osimertinib-induced AEs. The significant SOCs that met four criteria were neoplasms benign, malignant and unspecified (incl cysts and polyps) and congenital, familial and genetic disorders. Also, general disorders and administration site conditions, respiratory, thoracic and mediastinal disorders, and hepatobiliary disorders were significant SOCs that at least one of the four indices met the criteria.

**Table 2:** Signal strength of reports of Osimertinib at the System Organ Class (SOC) level in FAERS database.

System organ class (SOC)	Osimertinib cases reporting SOC	ROR (95% two-sided CI)	PRR (χ^2^)	IC (IC 025)	EBGM (EBGM 05)
General disorders and administration site conditions	6396	2.26 (2.17–2.35)*	1.51 (1824.95)	0.60 (0.55)*	1.51 (1.46)
Respiratory, thoracic and mediastinal disorders	1965	1.32 (1.26–1.39)*	1.26 (125.75)	0.34 (0.27)*	1.26 (1.20)
Neoplasms benign, malignant and unspecified (incl cysts and polyps)	1957	2.62 (2.49–2.75)*	2.32 (1595.83)*	1.21 (1.14)*	2.32 (2.21)*
Gastrointestinal disorders	1380	0.66 (0.62–0.70)	0.70 (214.75)	− 0.51 (− 0.59)	0.70 (0.66)
Nervous system disorders	1262	0.50 (0.47–0.53)	0.56 (568.56)	− 0.85 (− 0.93)	0.56 (0.52)
Skin and subcutaneous tissue disorders	1155	0.62 (0.58–0.66)	0.66 (240.22)	− 0.60 (− 0.69)	0.66 (0.62)
Cardiac disorders	1125	0.91 (0.86–0.97)	0.92 (8.18)	− 0.12 (− 0.21)	0.92 (0.87)
Vascular disorders	1065	0.60 (0.56–0.64)	0.64 (253.24)	− 0.64 (− 0.73)	0.64 (0.60)
Investigations	990	0.78(0.73–0.83)	0.80 (57.25)	− 0.33 (− 0.42)	0.80 (0.75)
Injury, poisoning and procedural complications	926	0.23 (0.21–0.24)	0.29 (2218.16)	− 1.77 (− 1.86)	0.29 (0.27)
Musculoskeletal and connective tissue disorders	786	0.56 (0.52–0.60)	0.59 (248.34)	− 0.75 (− 0.86)	0.59 (0.55)
Metabolism and nutrition disorders	770	1.00 (0.93–1.07)	1.00 (0.00)	− 0.01 (− 0.11)	1.00 (0.93)
Infections and infestations	760	0.60 (0.56–0.65)	0.63 (186.18)	− 0.67 (− 0.78)	0.63 (0.59)
Immune system disorders	677	0.63 (0.58–0.68)	0.65 (138.15)	− 0.62 (− 0.73)	0.65 (0.6)
Blood and lymphatic system disorders	573	1.09 (1.00–1.18)	1.08 (3.68)	0.11 (− 0.01)	1.08 (0.99)
Hepatobiliary disorders	487	1.74 (1.59–1.90)*	1.70 (144.90)	0.76 (0.63)*	1.70 (1.55)
Psychiatric disorders	417	0.27 (0.25–0.30)	0.30 (788.24)	− 1.74 (− 1.89)	0.30 (0.27)
Renal and urinary disorders	282	0.37 (0.32–0.41)	0.38 (302.13)	− 1.39 (− 1.56)	0.38 (0.34)
Congenital, familial and genetic disorders	251	4.10 (3.61–4.64)*	4.02 (570.66)*	1.98 (1.80)*	4.01 (3.54)*
Eye disorders	232	0.49 (0.43–0.56)	0.50 (118.88)	− 0.99 (− 1.18)	0.50 (0.44)
Reproductive system and breast disorders	72	0.15 (0.12–0.19)	0.15 (351.19)	− 2.70 (− 3.05)	0.15 (0.12)
Endocrine disorders	72	0.25 (0.20–0.32)	0.26 (158.35)	− 1.96 (− 2.30)	0.26 (0.20)
Ear and labyrinth disorders	49	0.35(0.26–0.46)	0.35 (59)	− 1.51 (− 1.93)	0.35 (0.27)
Surgical and medical procedures	31	0.08 (0.06–0.12)	0.09 (314.21)	− 3.55 (− 4.07)	0.09 (0.06)
Social circumstances	29	0.23 (0.16–0.33)	0.23 (76.47)	− 2.14 (− 2.68)	0.23 (0.16)
Product issues	14	0.03 (0.02–0.05)	0.03 (452.88)	− 5.04 (− 5.81)	0.03 (0.02)
Pregnancy, puerperium and perinatal conditions	2	0.01 (0–0.04)	0.01 (196.22)	− 6.62 (− 8.67)	0.01 (0.00)

*ROR* reporting odds ratio, *CI* confidence interval, *PRR* proportional reporting ratio, *χ*^*2*^ chi-squared, *IC* information component, *IC 025* the lower limit of 95% CI of the IC, *EBGM* empirical Bayesian geometric mean, *EBGM 05* the lower limit of 95% CI of EBGM.

*Indicates statistically significant signals in algorithm.

After excluding neoplasms benign, malignant and unspecified (incl cysts and polyps) which may cause by the disease progression, totally 68 significant disproportionality PTs conforming to the four algorithms simultaneously are shown in Table 3. According to the previous study of osimertinib, cardiotoxicity events, pneumonitis, eye disorders, and skin disease events are usually reported. In our study, long QT syndrome (PT: 10024803), cardiac failure (PT: 10007554), cardiomyopathy (PT: 10007636), platelet count decreased (PT: 10035528), paronychia (PT: 10034016), etc. are consistent with findings from clinical trials. Interestingly, unexpected significant AEs were uncovered in the label, including B-Raf proto-oncogene, serine/threonine kinase (BRAF) gene mutation (PT:10075648), volvulus (PT: 10047697), mechanical ileus (PT: 10051399), amylase increased (PT: 10002016), immobilisation syndrome (PT: 10084349), cerebral infarction (PT: 10008118), deep vein thrombosis (PT: 10051055), venous thrombosis limb (PT: 10061408)

**Table 3:** Signal strength of reports of Osimertinib at the Preferred Term (PT) level in FAERS database.

SOC	Preferred terms (PTs)	Osimertinib cases reporting PT	ROR (95% two-sided CI)	PRR (χ^2^)	IC (IC 025)	EBGM (EBGM 05)
Blood and lymphatic system disorders	Myelosuppression	79	15.53 (14.94–16.14)	9.68 (34,904.03)	3.26 (3.20)	9.57 (9.21)
Cardiac disorders	Cardiac failure	111	26.04 (23.54–28.82)	25.11 (9007.09)	4.52 (4.37)	24.36 (22.01)
	Pericardial effusion*	40	15.18 (13.63–16.92)	14.73 (4342.61)	3.80 (3.64)	14.47 (12.99)
	Cardiomyopathy*	37	2.84 (2.54–3.16)	2.78 (380.97)	1.46 (1.30)	2.77 (2.49)
	Cardiotoxicity	26	7.54 (6.59–8.64)	7.41 (1184.5)	2.83 (2.63)	7.35 (6.42)
	Cardiac dysfunction*	19	254.76 (216.08–300.36)	250.3 (35,659.35)	6.57 (6.33)	189.42 (160.66)
	Cardiac failure acute*	18	12.97 (11.18–15.05)	12.77 (1913.25)	3.56 (3.34)	12.58 (10.84)
	Long QT syndrome	8	3.63 (3.13–4.22)	3.59 (330.94)	1.81 (1.59)	3.58 (3.08)
	Ventricular dysfunction*	6	6.73 (5.77–7.87)	6.65 (777.17)	2.67 (2.44)	6.60 (5.65)
Congenital, familial and genetic disorders	Acquired gene mutation*	190	3.02 (2.58–3.54)	2.99 (205.95)	1.55 (1.32)	2.99 (2.55)
	EGFR gene mutation	39	3.47 (2.92–4.11)	3.44 (229.53)	1.74 (1.49)	3.43 (2.89)
	Gene mutation	14	7.93 (6.65–9.46)	7.85 (746.42)	2.87 (2.61)	7.78 (6.52)
	BRAF gene mutation*	8	3.94 (3.30–4.71)	3.91 (265.85)	1.92 (1.65)	3.90 (3.26)
Eye disorders	Keratitis	7	2.82 (2.34–3.40)	2.80 (128.8)	1.45 (1.17)	2.80 (2.32)
Gastrointestinal disorders	Stomatitis	123	2.97 (2.45–3.61)	2.95 (134.22)	1.52 (1.23)	2.95 (2.43)
	Volvulus*	7	4.72 (3.85–5.79)	4.69 (268.66)	2.15 (1.85)	4.67 (3.80)
	Mechanical ileus*	4	37.55 (30.14–46.78)	37.27 (2829.17)	4.64 (4.32)	35.6 (28.58)
General disorders and administration site conditions	Death	4,349	4.15 (3.33–5.16)	4.12 (190.86)	1.97 (1.64)	4.11 (3.30)
	Drug resistance	401	10.49 (8.39–13.10)	10.42 (664.14)	3.19 (2.86)	10.29 (8.24)
	Disease progression	177	2.84 (2.26–3.59)	2.83 (85.24)	1.44 (1.10)	2.83 (2.24)
Hepatobiliary disorders	Hepatic function abnormal*	126	17.93 (14.06–22.86)	17.82 (1040.3)	3.79 (3.43)	17.44 (13.68)
	Liver disorder*	81	14.98 (11.57–19.4)	14.91 (751.24)	3.55 (3.17)	14.64 (11.31)
Infections and infestations	Paronychia	84	3.33 (2.54–4.37)	3.32 (85.69)	1.64 (1.24)	3.31 (2.53)
	Pneumonia bacterial*	20	6.15 (4.63–8.18)	6.13 (204.53)	2.43 (2.01)	6.09 (4.58)
	Nail infection	8	4.26 (3.17–5.71)	4.24 (111)	1.95 (1.52)	4.22 (3.15)
	Infectious pleural effusion*	6	5.59 (4.17–7.5)	5.57 (167.81)	2.30 (1.87)	5.54 (4.13)
	Lymphangitis	5	14.76 (10.91–19.97)	14.71 (539.18)	3.44 (2.99)	14.45 (10.68)
Injury, poisoning and procedural complications	Radiation pneumonitis*	26	4.66 (3.43–6.34)	4.65 (116.7)	2.06 (1.6)	4.62 (3.40)
Investigations	Platelet count decreased	155	3.76 (2.75–5.13)	3.75 (80.27)	1.77 (1.31)	3.73 (2.74)
	Electrocardiogram QT prolonged	93	3.30 (2.41–4.53)	3.30 (62.19)	1.60 (1.14)	3.29 (2.40)
	Ejection fraction decreased	48	155.48 (110.19–219.39)	154.92 (4970.31)	4.91 (4.41)	129.27 (91.61)
	Blood creatine phosphokinase increased	45	9.50 (6.9–13.09)	9.47 (284.53)	2.91 (2.44)	9.37 (6.80)
	Carcinoembryonic antigen increased	37	40.00 (28.73–55.68)	39.86 (1333.32)	4.23 (3.74)	37.96 (27.26)
	Amylase increased*	13	6.09 (4.40–8.42)	6.07 (155.63)	2.37 (1.90)	6.03 (4.36)
	SARS-CoV-2 test negative	12	2.82 (2.03–3.91)	2.81 (42.00)	1.38 (0.90)	2.81 (2.02)
	Tumour marker increased	11	5.94 (4.03–8.73)	5.92 (105.64)	2.26 (1.69)	5.89 (4.00)
	BRAF V600E mutation positive*	6	18.92 (12.82–27.93)	18.88 (429.72)	3.43 (2.86)	18.45 (12.50)
	Myocardial necrosis marker increased*	5	4.73 (3.05–7.35)	4.73 (58.42)	1.93 (1.28)	4.70 (3.03)
Metabolism and nutrition disorders	Decreased appetite	332	9.17 (5.83–14.42)	9.16 (136.50)	2.62 (1.95)	9.06 (5.76)
	Hypophagia	36	5.38 (3.38–8.56)	5.38 (63.68)	2.04 (1.36)	5.34 (3.36)
Musculoskeletal and connective tissue disorders	Immobilisation syndrome*	4	6.82 (4.29–10.86)	6.81 (88.55)	2.30 (1.61)	6.76 (4.25)
Nervous system disorders	Taste disorder	41	5.77 (3.63–9.17)	5.76 (70.29)	2.12 (1.44)	5.72 (3.60)
	Cerebral infarction*	39	12.41 (7.32–21.05)	12.39 (144.35)	2.71 (1.93)	12.21 (7.20)
Respiratory, thoracic and mediastinal disorders	Interstitial lung disease	345	6.71 (3.89–11.59)	6.70 (62.57)	2.14 (1.33)	6.66 (3.85)
	Pleural effusion*	215	25.92 (14.91–45.05)	25.89 (300.99)	3.10 (2.28)	25.08 (14.43)
	Pneumonitis	179	9.91 (5.61–17.52)	9.90 (94.8)	2.43 (1.59)	9.79 (5.54)
	Lung disorder	163	26.89 (15.12–47.83)	26.87 (288.82)	3.04 (2.19)	26.00 (14.62)
	Pulmonary embolism*	133	12.16 (6.87–21.52)	12.15 (120.91)	2.58 (1.74)	11.98 (6.77)
	Respiratory failure	104	10.45 (5.76–18.95)	10.44 (92.68)	2.41 (1.54)	10.32 (5.69)
	Pneumothorax*	45	3.98 (2.2–7.21)	3.98 (24.44)	1.54 (0.67)	3.97 (2.19)
	Pulmonary alveolar haemorrhage	18	4.33 (2.25–8.33)	4.32 (22.88)	1.54 (0.58)	4.31 (2.24)
	Pulmonary toxicity	18	35.60 (18.24–69.46)	35.57 (289.1)	2.83 (1.85)	34.05 (17.45)
	Lung opacity	11	124.03 (58.79–261.67)	123.94 (841.01)	2.90 (1.81)	106.98 (50.71)
	Eosinophilic pneumonia*	8	6.24 (3.11–12.52)	6.24 (34.93)	1.80 (0.78)	6.20 (3.09)
	Hydrothorax*	7	5.42 (2.70–10.87)	5.42 (28.62)	1.69 (0.66)	5.39 (2.69)
	Pulmonary artery thrombosis*	5	10.37 (5.16–20.84)	10.36 (66.78)	2.17 (1.14)	10.24 (5.10)
Skin and subcutaneous tissue disorders	Nail disorder	67	13.81 (6.54–29.16)	13.80 (81.64)	2.21 (1.11)	13.57 (6.43)
	Onychoclasis	59	5.16 (2.45–10.86)	5.16 (23.32)	1.57 (0.47)	5.13 (2.44)
	Skin disorder	53	5.70 (2.71–11.99)	5.70 (26.9)	1.65 (0.55)	5.66 (2.69)
	Dermatitis acneiform*	43	232.51 (93.36–579.09)	232.38 (1063.33)	2.54 (1.23)	178.99 (71.86)
	Erythema multiforme*	38	7.25 (3.25–16.21)	7.25 (32.03)	1.71 (0.53)	7.19 (3.22)
	Onychalgia	13	8.45 (3.78–18.91)	8.45 (38.99)	1.81 (0.62)	8.37 (3.74)
	Ingrowing nail	12	11.46 (4.74–27.73)	11.46 (47.04)	1.79 (0.49)	11.31 (4.67)
	Nail discolouration	9	5.62 (2.33–13.54)	5.61 (18.82)	1.40 (0.10)	5.58 (2.31)
	Nail bed disorder	5	20.83 (8.57–50.65)	20.82 (91.89)	2.00 (0.70)	20.30 (8.35)
Vascular disorders	Deep vein thrombosis	72	14.19 (5.86–34.38)	14.19 (60.19)	1.88 (0.58)	13.95 (5.76)
	Venous thrombosis limb	12	28.18 (10.39–76.43)	28.17 (101.14)	1.80 (0.33)	27.21 (10.03)
	Thrombophlebitis migrans	9	14.35 (5.34–38.59)	14.34 (48.76)	1.64 (0.18)	14.10 (5.24)

*ROR* reporting odds ratio, *CI* confidence interval, *PRR* proportional reporting ratio, *χ*^*2*^ chi-squared, *IC* information component, *IC 025* the lower limit of 95% CI of the IC, *EBGM* empirical Bayesian geometric mean, *EBGM 05* the lower limit of 95% CI of EBGM.

*Emerging findings of Osimertinib associated AEs from FAERS database.

### Onset time of events

**Figure 1:**
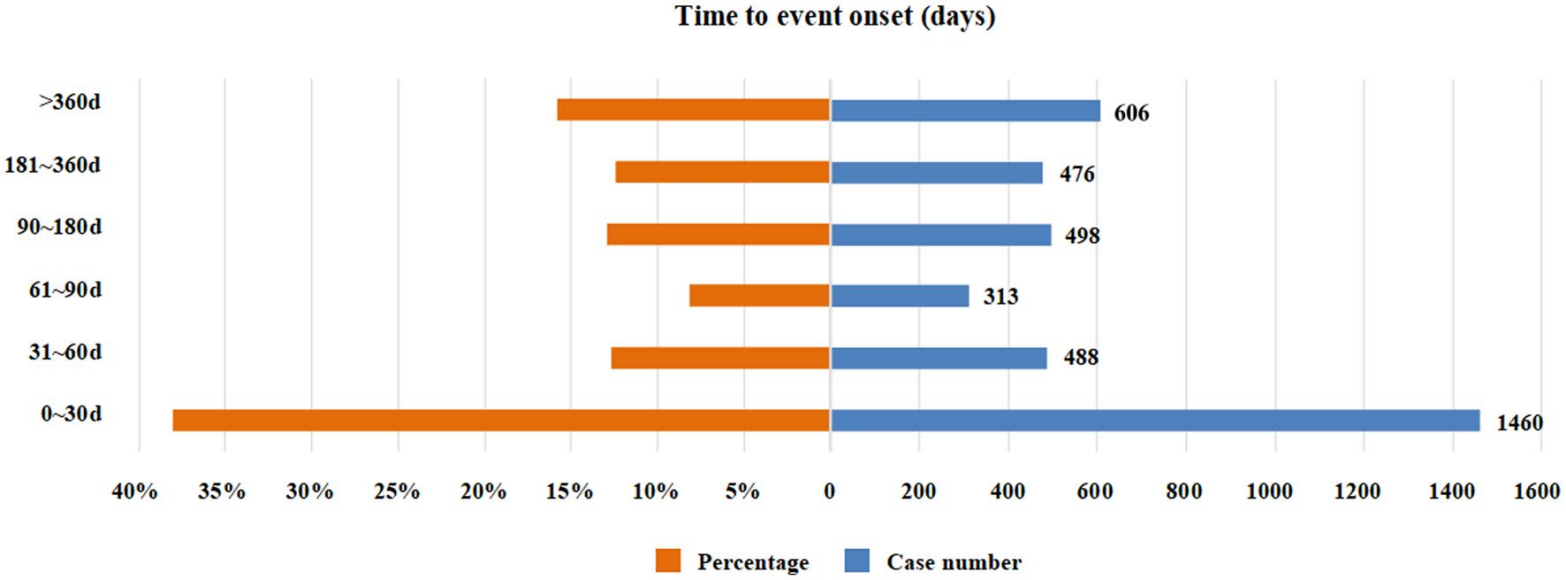
Time to onset of osimertinib-related AEs.

The onset times of osimertinib-associated AEs were collected from the database. Excluding false reports, totally 3841 AEs reported onset time. The median onset time was 58 days (interquartile range [IQR] 14–212 days). As shown in Fig. 1, results indicated that the onsets of osimertinib were general, cases may cover over a year. However, most of the cases occurred within the first month (n = 1460, 38.01%) after osimertinib initiation. However, the case ratios occurred in the 2 months (n = 488, 12.71%), 6 months (n = 498, 12.97%), and 12 months (n = 476, 12.39%) were similar, which reflected that AEs might occur at anytime within a year. Furthermore, AEs occurred after 1 year of osimertinib treatment with percentage of 15.78% (n = 606) as illustrated in our data.

## Conclusion

Our pharmacovigilance analysis of FAERS database revealed the safety signals of osimertinib and time to AEs onsets with osimertinib comprehensively and systematically. New and unexpected significant AEs as volvulus, hepatic function abnormal, and VTEs might also occur. High attention should be paid to common AEs included long QT syndrome, endocrine, pneumonitis, and cardiomyopathy. Completely monitoring and risk identification of all these AEs are suggested in all populations. Cohort studies and long-term clinical investigations are still needed to verified these results and to further comprehend the safety of osimertinib.

## Discussion

In previous studies, the research on osimertinib mostly focused on the mechanism, clinical trials, and literature analysis, etc., and few articles concentrated on the latest real-world research. Based on the largest samples of the real-world data, we collected and evaluated the pharmacovigilance of osimertinib in the post-market. The purpose is to analyze new and meaningful adverse reactions, to guide the update of summary of product characteristics (SmPC), and to provide a basis for clinical rational drug use.

The AEs of osimertinib occurred more commonly in females (55.45%) than in males (29.94%). This may be related to the increase of female patients with lung cancer, which leads to the increase of drug use opportunities. Studies have shown that females are more likely than males to have non-smoking lung cancer^13^. The causes of lung cancer in female may be related to the influence of estrogen^14^, environment^15^ and molecular factors^16^. Also, the study described a higher AEs proportion in elderly patients (36.98% patients > 65 years), which was consistent with the FLAURA and AURA clinical trials that the number of AEs leading to the dose adjustment (suspension or reduction) of the study drug was higher in subjects aged over 65 years^6^. With the increasing clinical application of osimertinib, it is important for clinicians to be alert to the AEs associated with osimertinib, especially in olderly patients. Early recognition of AEs is necessary because these effects can be life-threatening or lead to disease progression.

According to the disproportionality analysis, the most commonly reported and significant signals at SOC levels were general disorders and administration site conditions, and neoplasms benign, malignant and unspecified (incl cysts and polyps). These AEs included death, disease progression, malignant neoplasm progression, et al*.*, which were not recorded in the SmPC of osimertinib and might be related to the patient's own disease progression rather than the drug itself. Evidence suggested that approximately 38% of all dead lung cancer patients had single site metastasis and 19% had two or more metastases^17^. It might be irrational to judge whether tumor metastasis and tumor progression were caused by osimertinib only by ADR signals. Clinicians should distinguish whether tumor-related disease is caused by osimertinib. Besides, significant signals were observed in SOC of respiratory, thoracic, and mediastinal disorders. Relevant studies have pointed out that different EGFR-TKIs can cause respiratory toxicity, and the incidence of interstitial lung disease (ILD) is 0–5.3%, while there is no significant difference between osimertinib and the first two generations^18,19^. Blood and lymphatic system disorders which related to the laboratory abnormalities were commonly found in more than 20% of patients in clinical trial^6^. However, AEs included hepatobiliary disorders, congenital, familial and genetic disorders are not mentioned in the SmPC. We should pay attention to whether they have clinical significance to guide clinical medication.

Among all the AEs, the AEs involving respiratory system and cardiovascular system still deserve attention. The respiratory adverse reactions mentioned in the SmPC of osimertinib include cough, pneumonia, and ILD. This study also excavated signs of AEs such as pneumothorax, pleural effusion, and hydrothorax. And the most common AEs leading to discontinuation of osimertinib was ILD/pneumonitis. Fan et al.^18^ found that all EGFR-TKIs had drug-related toxicities included ILD, and the incidence of drug-related ILD in different EGFR-TKIs ranged from 0 to 5.3%. The mechanism of ILD may be different for third-generation EGFR-TKIs, because osimertinib induced ILD in patients who had no pulmonary toxicities during a prior treatment with first- or second-generation EGFR-TKI^19^. The mechanism of osimertinib induced respiratory toxicity is not clear, but it may be related to the inhibition of the maintenance of epithelial cells. Osimertinib changed the expression of cytokines by impairing the growth and migration of epithelial cells, resulting in inflammatory cell recruitment and lung tissue injury^20^. Although different EGFR-TKIs could cause respiratory toxicity, the AEs related to EGFR-TKI were usually tolerable and controllable. Risk factors, such as tobacco exposure, pre-existing lung fibrosis, and chronic obstructive pulmonary disease, indicate that lung inflammatory circumstances may worsen with EGFR-TKI treatment because of impaired epithelial healing of lung injuries^20^. Noonan et al.^21^ demonstrated NSCLC patients who had previously received chest radiotherapy or had a history of aspiration were more likely to have lung shadows or subpleural nodules after using osimertinib. Furthermore, a combination of drugs with or without radiotherapy can increase the risk of ILD^22^. Therefore, we speculated that patients with chronic lung injury in the past were the high-risk population of respiratory toxicity. Mamesaya et al.^23^ reported a case of a 38-year-old female patient with osimertinib-induced ILD after treatment with anti-PD1 antibody and speculated anti-PD1 therapies might be the risk factor of EGFR-TKI-induced ILD. Our study suggests that for patients with chronic respiratory diseases and combined PD-1/PD-L1 therapy, the pharmaceutical care of osimertinib should be strengthened.

Cardiotoxicity is accompanied by a history of treatment with antitumor drugs. Whether traditional chemotherapy^24^, new targeted therapy^25^ or immunotherapy^26^ can cause cardiac related AEs. Cardiac related AEs are a commom toxicity of TKIs existed in not only first and second generation but third generation^12,27^. Our study not only found out AEs signals of cardiac disorders like heart failure, QT interval prolongation, electrocardiogram QT prolonged, in the SmPC of osimertinib, but also excavated AEs like ventricular dysfunction, cardiology, cardiac dysfunction and cardiotoxicity that do not exist in the SmPC. In vitro, osimertinib not only inhibited EGFR but also human epidermal growth factor receptor-2 (HER2) at clinically relevant concentrations. The current mainstream view is that the inhibition of HER2 is the main reason for the cardiotoxicity of some antitumor drugs^28^. HER2 is essential for maintaining cardiac function, and HER2 inhibition is the main cause of cardiotoxicity for some antitumor drugs. Trastuzumab is a monoclonal antibody targeting HER2, whose cardiotoxicity increased by 2.45 times after treatment^29^. Perez et al.^30^ also found that osimertinib might lead to dose independent reversible myocardial injury by inhibiting erythroblastic leukemia viral oncogene B (commonly referred to as HER). Kunimasa et al.^31^ found severe osimertinib-associated cardiotoxicities with a higher frequency (4.1%) than previous studies. They also suggested that in the clinical application of osimertinib, attention should be paid not only to the QTc interval prolongation, but also to other cardiotoxicity. Although osimertinib was highly specific for EGFR, study showed that osimertinib has a greater inhibitory effect on HER2 than other EGFR-TKIs^32^. Furthermore, rates of QT prolongation, cardiac failure, and atrial fibrillation were found to be higher when osimertinib compared with other EGFR-TKIs in FAERS^12^. Based on all the facts we speculated that HER2 was the main cause of osimertinib cardiotoxicity. The risk factors of cardiotoxicity caused by antitumor drugs include age, potential heart disease, renal insufficiency, and the combination of other cardiotoxic drugs, while the risk of cardiotoxicity caused by EGFR-TKI is more closely related to the patient's cardiovascular history^33^. Thus, the early awareness of cardiotoxicities, monitoring for QT prolongation, managing symptoms of heart failure, and close follow-up, may enhance the benefits of therapy while taking osimertinib.

Excitingly, we found some unexpected and significant safety signals, which included BRAF V600E mutation positive, volvulus, hepatic function abnormal, and venous thromboembolism (VTE). *BRAF* is an important proto oncogene in human beings. About 15% of malignant tumors are related to *BRAF* mutation^34^. At present, there are many mutations in this gene, of which *BRAF* V600E is the most common accounts for 75–82% of *BRAF* mutations in cutaneous melanoma^35^. Different from *EGFR* and *ALK* gene mutations, *BRAF* V600 mutation is relatively rare in non-small cell lung cancer, about 2–3% of which are adenocarcinoma^36,37^. *BRAF* mutation will continuously activate the downstream MEK-ERK signal pathway and play a vital role in tumor growth, proliferation, invasion, and metastasis. There is no specific treatment for NSCLC patients carrying a *BRAF* mutation, even if in melanoma, BRAF inhibitors were demonstrated to prolong progression-free survival and survival^38^. Therefore, *BRAF* mutation represents strong tumor invasiveness. Therefore, we infer that *BRAF* mutation may be a signal reflecting disease progression.

Drug-induced liver injury is an important adverse effect of TKIs. In vitro, osimertinib was mainly eliminated by the liver and metabolized by Cytochrome P450 (CYP, P450) 3A4 and CYP 3A5. The main metabolic pathways are oxidation and dealkylation. In the AURA2 study, 1 patient developed drug induced liver injury (DILI) which was manifested by elevated serum aminotransferase levels (< 1%)^39^. Elevated liver transaminases (all grades) associated with EGFR-TKI use are seen in 25–55%, 27–38%, 10%, and 9% of patients treated with gefitinib, erlotinib, afatinib, and osimertinib, respectively. Severely (grade 3 or 4) elevated liver transaminases is found in 1% of patients treated with osimertinib^7^. The mechanism of liver injury caused by EGFR-TKI has not been fully elucidated. Some researchers believe that the liver toxicity of TKI drugs is related to the metabolism of their active metabolites, which can interfere with cellular molecules and thus affect cell function and death^40^. Autoimmune activation is also a mechanism by which TKIs cause hepatotoxicity^41^. Ivan Gonzalez et al.,^42^ reported that pericentral confluent necrosis and parenchymal collapse in liver biopsy after the patient treated with osimertinib and developed transaminitis of unclear etiology, which has been reported in other TKIs. Hirabayashi et al.^43^, reported a case that osimertinib induced hepatotoxicity after 15 days of treatment. Although there are not many case reports about liver injury, it is undeniable that it has become a class of AEs that needs to be paid enough attention to liver disorders are often improved with dose reduction or transient discontinuation of EGFR-TKIs, and concomitant use of hepatoprotective agents^17^.

Nowadays, there has been no association with volvulus and EGFR inhibition. The occurrence of volvulus is caused by a variety of reasons, and physiological or pathological factors are the predisposing factors. The risk factors of cecal volvulus included chronic constipation, distal colon obstruction, high-fiber diets, ileus, prior colonoscopy, and late pregnancy^44^. Constipation, a known risk factor for volvulus, was only found in 7 cases in clinical trial. Patil et al.^45^, reported 3 cases associating cecal volvulus with the 160 mg dose of osimertinib, and highlighted a potentially vital surgical complication associated with the 160 mg dose of osimertinib.

Tumor patients have a higher incidence of VTE than normal people. The mortality of tumor patients with VTE is a twofold increased mortality rate compared to cancer patients without VTE^46^. Accurate assessment of patients' risk of VTE can effectively prevent the occurrence of VTE events and reduce mortality. According to our study, osimertinib can also lead to deep vein thrombosis, venous thrombosis limb, and pulmonary embolism. A meta-analysis of venous thromboembolic events associated with VEGFR-TKIs found that the use of VEGFR-TKIs does not significantly increase the risk of VTEs, the risk of VTEs in cancer patients is mainly affected by tumor types, host factors, and concomitant usage of anticancer drugs^47^. Hotta et al.^48^, aimed to identify anticancer drugs and anticoagulants that can be used safely in combination, as accompanying study to an observational research on VTE incidence rates in lung cancer patients. And the study indicated that the PK of anticoagulants was not affected by co-administration of EGFR-TKIs (gefitinib, erlotinib, and afatinib). While, early diagnosis, appropriate treatment, and prevention are considered important measures to improve prognosis.

Results of our study showed that the median onset time was 58 days, and most cases occurred within the first month (n = 1460, 38.01%), after osimertinib initiation. In FLAURA and AURA, 49% of patients reported diarrhea with a median duration of 19 and 22 days and a median duration of 19 and 6 days, respectively. We also found that except for the first month, the probability of AEs within one year was similar. The median time of ILD or ILD like adverse reactions in the global population is 85 days. These results suggested that we should pay special attention to the AEs of patients in the first month and early recognition of AEs caused by osimertinib therapy could reduce the agony of patients which can be life-threatening.

Based on FAERS database, our study excavates and analyzes the adverse reaction signals of osimertinib, discusses the respiratory toxicity and cardiotoxicity related to osimertinib, and some other meaningful AEs, in order to provide some reference for improving the safety of clinical medication. FAERS database is a spontaneous reporting system. Due to its own limitations, there are phenomena such as underreporting, re-reporting, incomplete case information and so on. And lack of underlying disease and concomitant medication may affect the results. Moreover, media attention and recent publication of an adverse drug reaction in the literature might affect the reporting behaviors^49^. However, despite the facts that FAERS database has some limitations in pharmacovigilance studies, a comprehensive characterization of the AE signals from osimertinib and the discovery of some unexpected AE signals might provide foundation for further clinical studies of osimertinib. And the efficacy and safety of osimertinib still need to be continuously monitored.

## Methods

### Data source and collection

**Figure 2:**
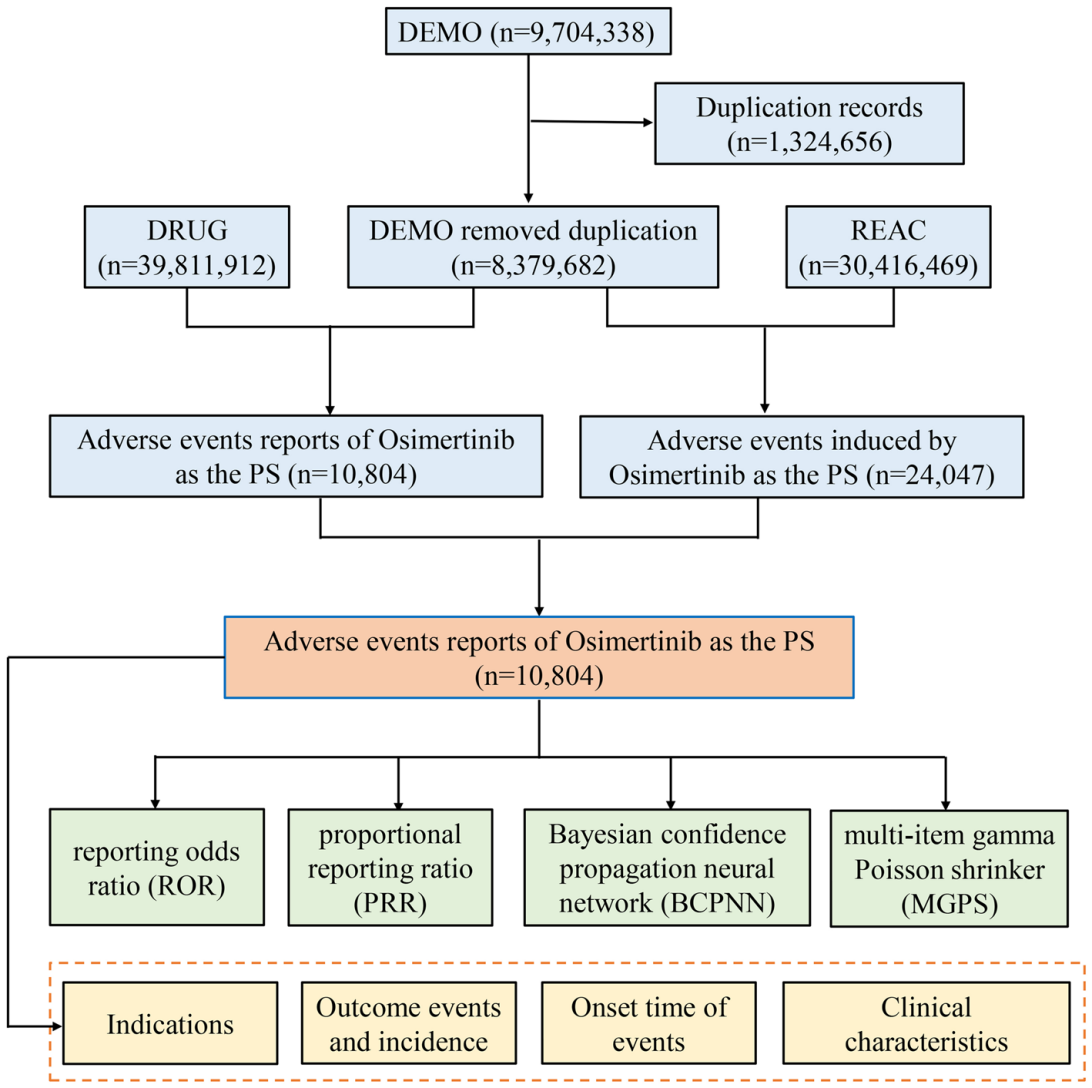
Flow diagram of this study (*DEMO* demographic and administrative information, *DRUG* drug information, *REAC* preferred terminology for adverse event, *PS* primary suspect drug).

We launched a pharmacovigilance study of osimertinib in the post-marketing setting using data covering the period from first quarter of 2016 to the fourth quarter of 2021 from the FAERS database. FAERS database is based on the International Safety Reporting Guidelines (ICH E2B) issued by ICH, and the adverse events are coded according to the Medical Dictionary for Regulatory Activities (MedDRA). Seven databases make up FAERS data files, including demographic and administrative information (DEMO), adverse drug reaction information (REAC), patient outcome information (OUTC), drug information (DRUG), drug therapy starts dates and end dates (THER), information on report sources (RPSR), and indications for use/diagnosis (INDI). We chose the latest FDA_DT with the same CASEID or selected the higher PRIMARYID when the CASEID and FDA_DT were the same to identify and remove duplicate reports. During the study period, totally 9,704,338 reports of osimertinib were gained from FAERS database. 8,379,682 case reports of osimertinib as the primary suspect (PS) drug after the exclusion of duplicates, and 10,804 AEs were associated with osimertinib (Fig. 2). All AEs reports of osimertinib were identified in system organ class (SOC) and PT levels. The codes of drugs reported in event include PS, secondary suspect drug (SS), concomitant (C), and interacting (I). Moreover, generic name (Osimertinib) and trade name (Tagrisso) were defined as target drugs in the DRUG file, and we chose the role_cod as PS.

### Statistical analysis

The association between osimertinib and AEs were determined by the reporting odds ratio (ROR), the proportional reporting ratio (PRR), the Bayesian confidence propagation neural network (BCPNN) and the multi-item gamma Poisson shrinker (MGPS) algorithms, which was based on the disproportionality analysis^50^. The equations and criteria for the four algorithms are described in Supplementary Table S1. The data that were chosen for analysis in our study were AE signals that met four algorithm standards. The novelty signals are identified as any significant AE which was not listed in package inserts^51^.

The onset time was defined as the interval between EVENT_DT (date of adverse event occurrence) and START_DT (start date for osimertinib use). Moreover, input errors (EVENT_DT earlier than START_DT) reports or inaccurate date entries were excluded. The time-to-onset was describled by median and interquartile ranges (IQR). MYSQL 8.0, Navicat Premium 15, Microsoft EXCEL 2016 and the GraphPad Prism 8 (GraphPad Software, CA, USA) were used to perform data processing and statistical analyses.

## Supplementary Information

Supplementary Information.

## Data Availability

The datasets generated during and/or analyzed during the current study are available from the corresponding author on reasonable request.
